# Ketamine use in the refractory and super refractory status epilepticus: a systematic review

**DOI:** 10.3389/fmed.2026.1959414

**Published:** 2026-09-21

**Authors:** Betsabé Troya-Córdova, Ricardo S. Pinto-Villalba

**Affiliations:** Universidad UTE, Facultad Ciencias de la Salud Eugenio Espejo, Carrera Atención Prehospitalaria y Emergencias, Grupo de Investigación en Medicina Prehospitalaria (GRIMPE), Quito, Ecuador

**Keywords:** epilectic seizures, epileptic drugs, ketamine, refractory status epilepctius (RSE), super refractory status epilepticus (SRSE)

## Abstract

**Background:**

Refractory status epilepticus (RSE) and super-refractory status epilepticus (SRSE) are associated with substantial morbidity, mortality, and intensive care requirements. Ketamine has emerged as a potential adjunct because of its N-methyl-D-aspartate receptor antagonism and relatively favourable haemodynamic profile.

**Objective:**

To evaluate the effectiveness and safety of ketamine in RSE and SRSE resolution.

**Methods:**

This systematic review followed PRISMA 2020 and was registered in PROSPERO (CRD42024625516). PubMed, Scopus, Web of Science, and Biblioteca Virtual en Salud were searched from inception to 20 November 2025. Two independent reviewers screened studies, extracted clinical, therapeutic, electroencephalographic, haemodynamic, and survival data, and assessed risk of bias using National Institutes of Health quality assessment tools. Meta-analysis was not performed because of substantial heterogeneity.

**Results:**

Twelve studies comprising 491 patients were included. Ketamine was generally administered as part of multimodal therapy after failure of several antiseizure medications and intravenous anaesthetics. Infusion rates ranged from 0.05 mg/kg/h to 10.5 mg/kg/h, and treatment duration ranged from 6 h to 29 days. Study-defined response rates following ketamine initiation ranged from 43.3% for complete electrographic seizure cessation to 100% for overall RSE/SRSE resolution; however, the highest estimates often reflected combined treatment rather than ketamine alone. Short-term or hospital survival ranged from 45.8% to 81.8%. Immediate haemodynamic deterioration appeared uncommon, although attribution was limited by baseline instability and concomitant therapy.

**Conclusion:**

Ketamine-containing regimens were associated with clinically relevant seizure reduction or cessation in RSE/SRSE, without a consistent signal of acute haemodynamic harm. However, the independent effect of ketamine on sustained seizure control, survival, and functional recovery remains uncertain. Prospective comparative studies using standardised dosing, timing, and outcome definitions are required.

**Systematic Review Registration:**

PROSPERO identifier CRD42024625516.

## Introduction

Status epilepticus (SE) is a time-dependent, potentially life-threatening neurological emergency characterised by prolonged or recurrent epileptic activity, with risk of neuronal injury, systemic deterioration, and death. Its clinical relevance lies not only in seizure duration, but also in the progressive loss of therapeutic responsiveness as the episode continues, requiring early recognition, stepwise treatment, and close monitoring. Reported incidence varies according to the population studied, diagnostic criteria, and healthcare setting, with estimates ranging approximately from 10 to 30 episodes per 100,000 person-years in Europe, although higher rates have been described in other series (1, 2). Overall mortality in SE is also variable and depends on factors such as age, aetiology, duration of the episode, presence of acute brain injury, and response to initial treatment (1–3).

Within the spectrum of SE, refractory status epilepticus (RSE) represents a more clinically severe form, defined by the persistence of clinical or electrographic seizures despite the appropriate use of a benzodiazepine and a second-line antiseizure medication (4). This condition occurs in a substantial proportion of patients with SE and is associated with a greater need for intensive care unit admission, mechanical ventilation, electroencephalographic monitoring, and the use of intravenous anaesthetic agents. Super-refractory status epilepticus (SRSE), in turn, is defined by the persistence or recurrence of SE after 24 h or more of anaesthetic treatment, including cases that recur during the tapering or withdrawal of sedation (2). This progression from SE to RSE and SRSE entails a substantial increase in therapeutic complexity and in the risk of unfavourable outcomes (2, 5).

Current guidelines recommend a stepwise treatment approach (3, 4, 6–8). In the initial phase, benzodiazepines constitute the treatment of choice; if epileptic activity persists, intravenous second-line antiseizure medications, such as levetiracetam, valproate, or fosphenytoin/phenytoin, are administered according to availability, patient profile, and clinical context. When these treatments fail, management of RSE usually requires intravenous anaesthetic agents, such as midazolam, propofol, or barbiturates, with the aim of suppressing clinical and electroencephalographic ictal activity. However, robust comparative evidence definitively establishing the superiority of one anaesthetic agent over another, or the optimal timing for introducing specific therapies in RSE and SRSE, remains lacking (8, 9).

The use of intravenous anaesthetic agents poses important challenges. Although these drugs may be effective in terminating epileptic activity, they are associated with respiratory depression, hypotension, need for mechanical ventilation, vasopressor support, and complications related to prolonged intensive care stay (5). This issue becomes particularly relevant in resource-limited settings, where access to intensive care units, continuous electroencephalographic monitoring, and ventilatory support may be restricted. Consequently, there is growing interest in therapeutic strategies capable of controlling refractory epileptic activity while offering a potentially more favourable haemodynamic and respiratory profile (5, 8).

In this context, ketamine has gained relevance as a therapeutic alternative in RSE and SRSE. Its mechanism of action as a non-competitive N-methyl-D-aspartate (NMDA) receptor antagonist provides a specific pathophysiological rationale, as prolonged SE is associated with progressive internalisation of GABA-A receptors and functional enhancement of NMDA-mediated excitatory neurotransmission (10). This phenomenon may contribute to the progressive loss of response to GABAergic drugs while also supporting the potential benefit of anti-NMDA agents in refractory or prolonged phases of SE. In addition, unlike other anaesthetic agents, ketamine tends to better preserve cardiovascular and respiratory stability, which may represent a clinical advantage in critically ill patients (9).

Despite this pathophysiological rationale and the progressive increase in its clinical use, evidence regarding ketamine in RSE and SRSE remains heterogeneous. Most available data derive from observational studies, retrospective case series, clinical registries, and reports with variable sample sizes (7, 11–13). Furthermore, ketamine is usually administered as part of multimodal therapeutic regimens, in combination with benzodiazepines, intravenous anaesthetic agents, and multiple antiseizure medications (14, 15). This makes it difficult to distinguish the overall clinical response to ketamine-containing multimodal treatment regimens from an effect independently attributable to ketamine.

In this context, the present systematic review aimed to evaluate the effectiveness and safety of ketamine in the treatment of refractory and super-refractory status epilepticus. Specifically, it sought to describe the clinical, aetiological, therapeutic, and electroencephalographic characteristics of patients treated with ketamine.

## Methods

### Protocol and registration

This study was conducted following the Preferred Reporting Items for Systematic Reviews and Meta-Analyses (PRISMA) 2020 guidelines. The research protocol, as well as the full search criteria were uploaded and registered in PROSPERO (CRD42024625516).

### Eligibility criteria

Eligibility criteria were established following the PICOS framework (Table 1). The language of the included studies was limited to English and Spanish; other languages were excluded manually during the filtering process. All available studies from the inception of the databases to 20 November 2025 were assessed for eligibility.

**Table 1:** Summary of inclusion and exclusion criteria using the PICOS framework.

Inclusion	Exclusion
Patient	
Adults, (≥18 years old) with suspected or confirmed refractory status epilepticus (RSE) and super-refractory status epilepticus (SRSE).	Paediatric population (age <18 years old), animal models, or other neurological diseases which one's mimics seizures or non-epileptic seizures
Intervention	
Ketamine use alone or with other non-convulsive agents or antiseizure medications (ASMs)	Non-specific or non-evaluable use of ketamine
Comparison	
None	None
Outcome	
Effectiveness of ketamine in resolving refractory status epilepticus (RSE) and super-refractory status epilepticus (SRSE).	
Study design	
Original studies	Literature reviews Letters to the editor Animal studies

### Information sources

Studies were identified in PubMed, Scopus, Web of Science and Biblioteca Virtual en Salud (BVS); the references of selected articles were also screened. No filters were used. BVS includes scientific literature from Latin America and the Caribbean, mainly written in Spanish; inclusion of such studies is limited in most systematic reviews that only consider the English language and therefore neglect literature from the Latin-American region.

### Search

A full search strategy was established, which can be found in the aforementioned PROSPERO registration. Search terms were tailored based on the PICOS framework and the eligibility criteria; the following key terms, with variations, were used: refractory status epilepticus, super refractory status epilepticus, ketamine, treatment, management, therapy.

### Study selection

Two blinded reviewers carried out an independent and standardized evaluation of the selected studies using Mendeley Reference Manager. Deduplication was performed automatically using Mendeley Reference Manager and two stages of screening were performed. During the first stage, reviewers screened titles and abstracts, while the second stage involved full-text review; after these, data of the selected studies was extracted in a spreadsheet. Any discrepancies between the reviewers were resolved by discussion and mutual consensus.

### Data items and collection process

For data extraction, we developed a standardized spreadsheet including the following variables: author; year of publication; type of study; number of participants; age; aetiology. Aetiology was classified according to the International League Against Epilepsy (ILAE) categories (i.e., structural, genetic, infectious, metabolic, immune, or unknown). Anoxic/post-cardiac arrest aetiology was recorded as an additional category because it was frequently reported across the included studies. Classification of status epilepticus [refractory status epilepticus (RSE) and super-refractory status epilepticus (SRSE)]; seizure type; electroencephalogram response; use of antiseizure medications (ASMs) or anaesthetic agents; need for vasopressors; time until the onset of ketamine; time of ketamine use; ketamine dosage; treatment effectiveness; effectiveness attributable to ketamine; hospital survival. If data was missing or inconsistent, we attempted to contact the authors; if they did not answer after repeated attempts, we considered it lost data. Due to the high information heterogeneity between reviewed studies, we could not perform a meta-analysis or establish a formal relationship between ketamine onset and clinical resolution.

### Risk of bias in individual studies

Both reviewers assessed the risk of bias independently using the Study Quality Assessment Tools from the National Institutes of Health (NIH). Studies were graded as either minimally low, moderately low, or high risk of bias. If answering yes in less than 50% of the questions, the study was graded as poor and hence had a high risk of bias; if between 50% and 79%, it was graded as fair and hence had a moderately low risk of bias; and if 80% or more, it was graded as good and hence had a minimally low risk of bias. This methodology has been used previously in other systematic reviews (16–18).

## Results

The searches from the inception of the databases to 20 November 2025 yielded a total of 1,948 results. Following the removal of duplicate entries, a subset of 1,003 articles remained for further evaluation during the screening phase. The complete process of article screening can be found in Figure 1.

**Figure 1:**
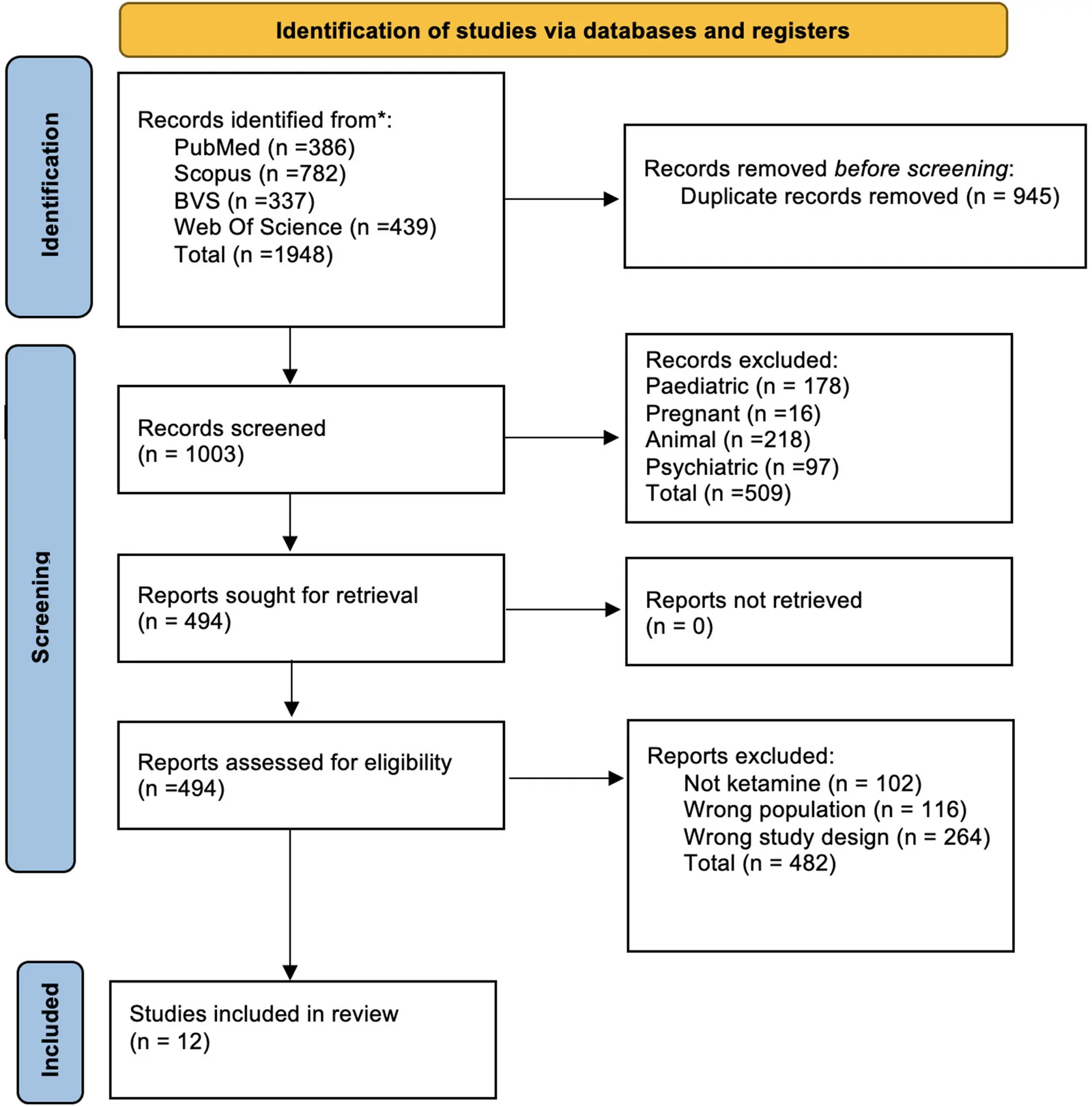
PRISMA flow chart.

A total of 12 studies were finally included (10–15, 19–24); these studies, with their year of publication, design, and bias assessment, are shown in Table 2.

**Table 2:** Risk of bias assessment.

*N*	Study	Year	Study design	Risk of bias
1	Sabharwal et al. (14)	2015	Cohort study	Moderately low risk
2	Basha et al. (19)	2015	Cross-sectional	Moderately low risk
3	Synowiec et al. (20)	2013	Case series	Low risk
4	Alkhachroum et al. (21)	2020	Cohort study	Moderately low risk
5	Gaspard et al. (15)	2013	Cohort study	Moderately low risk
6	Dericioglu et al. (22)	2020	Case series	Low risk
7	Srinivas et al. (23)	2022	Cohort study	Moderately low risk
8	Höfler et al. (24)	2016	Cohort study	Moderately low risk
9	García-Ruiz et al. (13)	2024	Cross-sectional	Moderately low risk
10	Harnicher et al. (12)	2024	Cohort study	Moderately low risk
11	Machado et al. (10)	2022	Cohort study	Moderately low risk
12	Caranzano et al. (11)	2022	Cohort study	Moderately low risk

The included studies comprised 491 reported patients with refractory status epilepticus (RSE) or super-refractory status epilepticus (SRSE). A sex-disaggregated synthesis was not possible because sex was either incompletely reported or presented inconsistently across studies. Similarly, age was reported using different summary measures; study-level mean or median ages ranged from 22 to 86 years.

Despite differences in reporting, structural causes were the most frequently identified aetiology, accounting for 27.7% of the reported cases, followed by anoxic or post-cardiac arrest brain injury (24.2%) and infectious causes (14.5%). However, aetiological definitions differed across studies, and some categories were not necessarily mutually exclusive. These proportions should therefore be interpreted as descriptive estimates rather than directly comparable pooled frequencies.

Variability was also evident in the treatment administered before ketamine. Where reported, patients had received between 0 and 7 antiseizure medications (ASMs) before ketamine initiation, while three studies did not provide this information. The timing of ketamine initiation was similarly heterogeneous, ranging from approximately 24 h to 11 days after the diagnosis of status epilepticus. These differences in baseline characteristics and previous treatment exposure provide important context for the marked variability subsequently observed in ketamine administration and clinical outcomes. The complete characteristics of patients in the included studies are presented in Table 3.

**Table 3:** Characteristics of patients in the included studies.

Study	Sample size	Population	Age, years	Main reported aetiologies	Antiseizure medications before ketamine	Time to ketamine initiation
Sabharwal et al.	67	SRSE	Mean 58; median 62	Toxic/metabolic, 18; anoxic/post-cardiac arrest, 13; unknown, 11; structural, 10; infectious, 10; autoimmune, 3; genetic, 2	Not reported	Not reported from SE onset
Basha et al.	11	RSE	Mean 54; range 33–68	Structural, 5; unknown, 2; infectious, 1; autoimmune, 1; toxic/metabolic, 1; anoxic, 1	1–5 ASMs	Mean 5.4 days; range 24 h–11 days
Synowiec et al.	11	RSE	Mean 52; range 22–82	Infectious, 7; structural, 3; toxic/metabolic, 1	7 ASMs and IV anaesthetics	Mean 5 days; range 1–11 days
Alkhachroum et al.	68	SRSE	Mean 53 ± 18	Structural, 17; infectious, 8; anoxic/post-cardiac arrest, 18; unknown, 25	Mean 2 ± 1 ASMs	Median 2 days; range 1–4.5 days
Gaspard et al.	60	RSE	Median 24	Toxic/metabolic, 2; anoxic/post-cardiac arrest, 7; unknown, 34; structural, 13; infectious, 4	6 ASMs	Median 9 days; range 0–122 days
Dericioglu et al.	7	RSE/SRSE	Median 66; range 44–86	Structural, 2; infectious, 5	2–5 ASMs	2–7 days from SE onset
Srinivas et al.	81	RSE	Mean 54.7 ± 15; median 55 (IQR 16)	Structural, 33; anoxic brain injury, 17; unknown, 11; toxic/metabolic, 5; infectious, 10; autoimmune, 5	Mean 3.0; range 1–6 ASMs	Mean 2.8 days; range 0.5–34 days
Höfler et al.	42	RSE/SRSE	Median 67 (IQR 59–72)	Anoxic/post-cardiac arrest, 14; structural, 10; unknown, 7; infectious, 4; genetic, 7	Median 3 ASMs	Median 3 days; range 2–7
García-Ruiz et al.	58	RSE/SRSE	Mean 60.2 ± 15.7	Anoxic/post-cardiac arrest, 24; structural, 17; metabolic/toxic, 6; unknown, 6; infectious, 5	Median 5 ASMs	Median 37 h from SE onset
Harnicher et al.	51	RSE/SRSE	Median 58 (IQR 29)	Toxic/metabolic, 7 infectious, 10; structural, 15; anoxic/post-cardiac arrest, 15; unknown, 4	Mean 4.4 ± 1.6 ASMs	Median 44.8 h from SE onset
Machado et al.	24	RSE/SRSE	Mean 51 ± 14.8	Anoxic/post-cardiac arrest, 10; structural, 9; unknown, 1; infectious, 4	Not reported as a cohort summary	Approximately 1 day from SE diagnosis
Caranzano et al.	11	SRSE	Mean 48 ± 22; median 46	Unknown, 3; infectious, 3; structural, 2; metabolic, 2; autoimmune, 1	Not reported	Median 4 days from SE onset

### Ketamine administration in RSE and SRSE

Consistent with the heterogeneity in previous treatment exposure, ketamine dosing and duration varied substantially across studies. Continuous infusion rates ranged from 0.05 mg/kg/h to 10.5 mg/kg/h. Treatment duration ranged from 6 h to 29 days, reflecting considerable differences in clinical severity, treatment protocols, and therapeutic objectives.

Ketamine was not generally used as monotherapy but as part of multimodal treatment regimens. Concomitant ASMs or intravenous anaesthetics included midazolam, propofol, pentobarbital, thiopental, lorazepam, valproate, lacosamide, levetiracetam, and other antiseizure medications. Its use also extended across different electroclinical presentations, including non-convulsive status epilepticus, generalised tonic–clonic status epilepticus, focal motor seizures, focal non-convulsive seizures, and mixed seizure patterns. The coexistence of these variations in seizure phenotype, treatment sequence, concomitant medications, ketamine dose, timing of initiation, and infusion duration limited direct comparisons between studies. Consequently, the available data did not allow meaningful stratification by seizure type or treatment regimen, nor did they permit the identification of an optimal dose, treatment duration, or therapeutic window for ketamine.

Clinical outcomes must be interpreted within this context. Where reported, short-term or in-hospital survival ranged from 45.8% to 81.8%. However, survival was assessed at different time points, including hospital discharge and fixed follow-up intervals. Therefore, these estimates should not be interpreted as directly comparable measures of treatment effectiveness. Further details regarding ketamine administration, concomitant therapy, and clinical outcomes are presented in Table 4.

**Table 4:** Ketamine administration, electroclinical findings, and outcomes.

Study	Ketamine regimen	Treatment duration	Main concomitant therapies	Seizure phenotype and EEG assessment	Haemodynamic and safety findings	Survival
Sabharwal et al.	Continuous infusion, 1.5–10.5 mg/kg/h equivalent to 25–175 µg/kg/min	1–29 days	Propofol in 67/67 patients; other ASMs according to clinical requirements	SRSE resolution assessed clinically and with continuous EEG; detailed seizure phenotypes were not stratified	Vasopressors used in 53/67 patients (79%)	41/67 (61.2%)
Basha et al.	Continuous infusion 1–5 mg/kg/h	2–27 days	Midazolam, 8/11; propofol, 4/11; pentobarbital, 3/11	Mixed focal, non-convulsive, and secondarily generalised RSE; a generalised theta-to-beta EEG pattern appeared in 5 patients and was associated with response in 4	No haemodynamic events nor ketamine-specific adverse events identified.	8/11 (72.7%)
Synowiec et al.	Continuous infusion 0.45–2.1 mg/kg/h	1–20 days	Propofol, midazolam, pentobarbital, lorazepam, and multiple ASMs	Generalised and focal convulsive or non-convulsive RSE; infusion titrated according to continuous EEG and electroclinical seizure control	Six of seven patients requiring vasopressors were weaned during ketamine infusion; no acute ketamine-related adverse effects	9/11 (81.8%)
Alkhachroum et al.	Mean infusion 2.2 ± 1.8 mg/kg/h; range 0.2–10 mg/kg/h	Median 2 days; range 1–4	Midazolam, 68/68; propofol, 36/68; pentobarbital, 10/68	Focal and generalised SRSE; ≥50% reduction in seizure burden and complete cessation evaluated within 24 h	Mean arterial pressure remained stable, with reduced vasopressor requirements 8% over time	37/68 (54.4%)
Gaspard et al.	Infusion median 2.75 mg/kg/h, range 0.05–10 mg/kg/h	6 h –27 days	Ketamine was part of regimens containing 2–12 medications, including midazolam, propofol, pentobarbital, and thiopental	Predominantly focal non-convulsive SE; control required cessation of clinical and electrographic ictal activity without recurrence during the ICU stay	Vasopressors used in 52/60 patients (86.7%); ketamine was discontinued for suspected adverse events in five cases, including tachyarrhythmias	34/60 (56.7%)
Dericioglu et al.	Continuous infusion 1–5 mg/kg/h	3–24 days	Midazolam, 6/7; propofol, 2/7; thiopental, 1/7	Refractory or super-refractory NCSE; control or improvement assessed through prolonged video-EEG monitoring	No haemodynamic events nor ketamine-specific adverse events identified.	5/7 (71.4%)
Srinivas et al.	Median continuous infusion 2.43 mg/kg/h; maximum 5.55 mg/kg/h	Mean 5 days; range 1–29	Midazolam, propofol, pentobarbital, and lorazepam	NCSE, 38.3%; focal SE, 33.3%; convulsive SE, 25.9%; myoclonic SE, 13.6%; successful response required electrographic cessation with EEG recovery	Vasopressor requirement increased in 9/81, decreased in 11/81, and remained unchanged in 61/81; volume overload in 23/81 and pulmonary oedema in 15/81	In-hospital survival not reported
Höfler et al.	Median infusion rate 2.39 mg/kg/h; median maximum rate 2.55 mg/kg/h	Median 4 days; range 2–7	Multiple ASMs and anaesthetics; propofol in 17/42, including concurrent administration in six	Predominantly comatose NCSE, 28/42; myoclonic, 6/42; tonic–clonic, 6/42; focal motor, 2/42; response assessed clinically and electrographically within 72 h	No haemodynamic events nor ketamine-specific adverse events identified.	23/42 (54.8%)
García-Ruiz et al.	Mean infusion 1.86 mg/kg/h; mean maximum dose 2.74 mg/kg/h	Median approximately 3–4 days	Midazolam, propofol, and multiple ASMs	Prominent motor symptoms in 46/58; response defined as seizure disappearance without recurrence after ketamine was the final medication introduced	No haemodynamic events nor ketamine-specific adverse events identified.	27/58 (46.6%)
Harnicher et al.	Median average rate 1.85 mg/kg/h equivalent to 30.8 µg/kg/min	38.2–40.2 h	Lorazepam, lacosamide, propofol, midazolam, valproate, and other ASMs	Convulsive SE, 29.4%; NCSE, 45.1%; both, 25.5%; ≥50% reduction in seizures: 84% at 24 and 90.5% at 48 h; complete cessation 43.3%; recurrence 20%	Vasopressor initiated within 2 h in 2/51; antihypertensive treatment in 1/51	26/51 (51.0%)
Machado et al.	Initial infusion approximately 1.2 mg/kg/h; maximum 2.2 mg/kg/h in responders and 2.4 mg/kg/h in non-responders	Not reported as a cohort summary	Midazolam, propofol, lacosamide, levetiracetam, pentobarbital, and other ASMs	NCSE, 11/24; convulsive SE, 13/24; focal motor or generalised presentations; ketamine-induced beta activity was associated with response	No haemodynamic events nor ketamine-specific adverse events identified.	11/24 (45.8%)
Caranzano et al.	Median 5 mg/kg/h; range 2.5–15 mg/kg/h	Median 2 days; range 1–16	Midazolam, 9/11; propofol, 2/11; thiopental, 1/11; phenobarbital, 4/11; other ASMs	Comatose NCSE, 8/11; generalised tonic–clonic RSE, 1/11; focal impaired-awareness RSE, 2/11; Crisis suppression in EEG	No haemodynamic events nor ketamine-specific adverse events identified.	7/11 (63.6%)

### Haemodynamic outcomes

In the management of RSE/SRSE, one of the main concerns is haemodynamic instability due the adverse effects of ASMs or ketamine combination. Given that ketamine was administered to critically ill patients who frequently received multiple anaesthetic and antiseizure medications, haemodynamic outcomes were particularly difficult to interpret. Five studies reported vasopressor use or other haemodynamic events, but baseline cardiovascular instability and vasopressor exposure before ketamine initiation were not consistently distinguished from events occurring after treatment.

Sabharwal et al. reported vasopressor use in 53 of 67 patients (14), while Gaspard et al. reported vasopressor exposure in 52 of 60 treated patients (15). These findings primarily describe the overall haemodynamic support required during the management of severe RSE or SRSE and cannot be interpreted as adverse effects directly caused by ketamine. Alkhachroum et al. reported a reduction in vasopressor requirements in approximately 8% of patients. While Srinivas et al. observed increased vasopressor requirements in 9 of 81 patients, decreased requirements in 11 of 81, and no change in 61 of 81 (23). Harnicher et al. reported vasopressor initiation within two hours of ketamine administration in 2 of 51 patients and antihypertensive treatment in 1 of 51 (12).

Although these findings dońt suggest marked haemodynamic deterioration immediately following ketamine administration, the heterogeneity of definitions, assessment periods, baseline instability, and concomitant therapies prevented data pooling. Accordingly, no direct relationship between ketamine administration and haemodynamic improvement or deterioration could be established. The reported haemodynamic outcomes are summarised in Table 4.

### Treatment response and attribution to ketamine

The interpretation of ketamine efficacy was similarly affected by methodological and clinical heterogeneity. Treatment response was generally defined as clinical or electrographic cessation of RSE or SRSE after ketamine initiation. In several studies, response was considered more directly associated with ketamine when it was the final medication introduced before seizure resolution (Table 5).

**Table 5:** Overall RSE or SRSE response to ketamine.

Study	Analysis population	Overall RSE/SRSE resolution	Response definition and interpretation
Sabharwal et al.	67	91.0%	Global resolution of SRSE in patients treated with propofol-ketamine combination.
Basha et al.	11	72.7%	Ketamine response required seizure cessation without recurrence when ketamine was the final medication added.
Synowiec et al.	11	100%	Cessation/resolution of refractory status epilepticus during ketamine use.
Alkhachroum et al.	68	≥50% seizure-burden reduction in 80.9%; complete cessation in 63.2%	≥50% reduction in ictal burden or complete cessation of SRSE after initiation of ketamine.
Gaspard et al.	60	56.7%	A likely response required permanent control within 24 h when ketamine was the final drug added.
Dericioglu et al.	7	71.4%	Resolution of NORSE when ketamine was the final medication added.
Srinivas et al.	81	60.5%	Ketamine-associated cessation required ketamine to be the final IV anaesthetic drug before electrographic seizure termination.
Höfler et al.	42	64.3%	Response was defined as clinical or electrographic cessation within 72 h.
García-Ruiz et al.	58	56.9%	Good response required seizure disappearance without recurrence when ketamine was the final medication administered.
Harnicher et al.	51	≥50% seizure reduction at 24 h in approximately 84%; complete cessation in 43.3%	The primary endpoint included reduction in seizure and interictal continuum burden. Complete cessation represented the stricter endpoint.
Machado et al.	24	50.0%	Responders achieved electrographic seizure cessation without recurrence; beta activity induced after ketamine was associated with response.
Caranzano et al.	11	Complete control 63.6%; sustained control after withdrawal 27.3%	Control during infusion was distinguished from persistent seizure freedom after ketamine discontinuation.

Electroencephalographic criteria varied across studies and included complete seizure suppression, disappearance of ictal activity, resolution or improvement of periodic discharges, electroclinical cessation, improvement in EEG patterns, and a reduction of at least 50% in seizure burden. As a result, the reported response rates reflected different levels of therapeutic effect, ranging from partial reduction in ictal activity to complete and sustained seizure cessation Table 4.

Based on the definitions used by the individual studies, response rates following ketamine initiation ranged from 43.3% for complete electrographic seizure cessation to 100% for overall RSE/SRSE resolution. These estimates represent outcomes observed during ketamine-containing regimens and should not be interpreted as the independent efficacy of ketamine.

However, the highest response rate was reported in a cohort treated with combined propofol and ketamine and should therefore be interpreted as the effectiveness of the combined regimen rather than ketamine alone.

Overall, the included studies consistently reported clinical or electrographic improvement in a relevant proportion of patients with RSE or SRSE. Nevertheless, the predominance of retrospective uncontrolled designs, variability in response definitions, differences in ketamine administration, and frequent use of concomitant antiseizure and anaesthetic medications limited comparisons across studies. Consequently, although ketamine-containing multimodal regimens were associated with a potentially favourable therapeutic response, the independent contribution of ketamine itself to seizure resolution could not be established.

## Discussion

This systematic review found that ketamine-containing regimens were associated with clinical or electrographic improvement in patients with refractory status epilepticus (RSE) or super-refractory status epilepticus (SRSE). However, response estimates varied according to the endpoint applied, from partial seizure-burden reduction to complete and sustained electrographic cessation. Ketamine-containing multimodal regimens therefore represent a plausible rescue strategy; however, the available evidence does not establish an independent ketamine effect or superiority over conventional intravenous anaesthetics.

This uncertainty is not unique to ketamine. Evidence supporting midazolam, propofol, pentobarbital, and thiopental is predominantly observational. In a systematic review of 192 patients, overall mortality was 48% and 29% returned to premorbid functional status. Pentobarbital reduced acute treatment failure and breakthrough seizures compared with midazolam or propofol, but caused more hypotension, without differences in mortality or functional recovery (6). A subsequent randomised comparison of propofol and barbiturates enrolled 24 participants and was underpowered to establish comparative effectiveness (25).

Recent evidence has not resolved this uncertainty. In an international cohort of 386 RSE episodes, a second anaesthetic was required in 26% of patients receiving midazolam and 35% receiving propofol, with comparable outcomes (7). Likewise, a systematic review of 66 studies involving 1,637 patients found differences among intravenous anaesthetics in short-term treatment failure and hypotension, but the initial agent was not independently associated with mortality (4).

This context is essential when interpreting the 45.8–81.8% short-term survival observed in our review. These estimates overlap with outcomes reported in RSE and SRSE treated without ketamine, but differences in aetiology, severity, follow-up, and inclusion of post-anoxic cases prevent direct comparison and do not support a survival advantage.

The distinction between seizure control and neurological recovery is equally important. An individual-participant meta-analysis of non-anoxic SRSE reported in-hospital mortality of 24.1%, while only 26.8% of patients had no or slight disability at discharge (26). Thus, electrographic suppression should not be considered equivalent to clinical recovery. In our review, the highest efficacy estimates generally reflected immediate or eventual seizure control, whereas sustained control after treatment withdrawal was less frequently demonstrated. Outcomes should therefore distinguish seizure-burden reduction, complete cessation, sustained control, survival, and functional recovery.

Ketamine should also be considered among other rescue interventions, including prolonged anaesthetic coma, additional antiseizure medications, ketogenic diet, immunotherapy, neurostimulation, and epilepsy surgery (27, 28). However, evidence for most approaches is similarly limited. Previous ketamine reviews reported favourable seizure-control rates and suggested greater effectiveness with earlier administration, but identified the same limitations observed here, observational designs, heterogeneous dosing, variable treatment latency, inconsistent EEG endpoints, and frequent coadministration of other anaesthetics (29, 30).

Haemodynamic findings also require caution. Conventional GABAergic anaesthetics frequently cause hypotension; this was reported in 68% of pentobarbital-treated patients compared with 36% receiving midazolam or propofol at Claassen et al., study (6). In our review, marked deterioration immediately after ketamine initiation appeared uncommon. Nevertheless, baseline shock, concurrent anaesthetic reduction, fluid therapy, and vasopressor adjustments were insufficiently reported, preventing attribution of haemodynamic improvement to ketamine.

Despite increasing clinical use, no adequately powered randomised trial has established ketamine efficacy specifically in RSE or SRSE. The limited randomised evidence available has largely emerged from paediatric settings where their results are not applicable to adults' population: KETASER01 trail was terminated after enrolling only 10 children, and two additional trials were withdrawn before enrolment (31, 32). A 2025 paediatric trial reported greater seizure cessation with ketamine plus midazolam than with midazolam alone in generalised convulsive status epilepticus, but these patients had not progressed to RSE or SRSE limiting the applicability of these findings to the adult population (33). This highlights the need of further randomised clinical trials in adult patients. However, the ongoing phase III KESETT trial may clarify the role of earlier ketamine administration in benzodiazepine-refractory status epilepticus (34).

Overall, ketamine-containing regimens achieved seizure-control rates overlapping those of conventional intravenous anaesthetics, while immediate haemodynamic deterioration appeared uncommon. However, current evidence does not show that ketamine improves survival, functional recovery, or sustained seizure control compared with other rescue therapies. The main contribution of this review is to identify a persistent attribution problem, seizures may improve after ketamine introduction, but available studies rarely establish whether this results from ketamine, concomitant therapy, or the evolution of the underlying condition. Future comparative studies should use standardised protocols and hierarchical outcomes. Until such evidence is available, ketamine may be considered a rational adjunctive therapy, although its independent effectiveness remains uncertain and its use should be approached with caution.

## Limitations

Several limitations should be acknowledged. The available evidence was predominantly derived from retrospective observational studies and small case series, which were susceptible to selection bias, incomplete reporting, and confounding by indication. Ketamine was generally administered after failure of multiple antiseizure medications and intravenous anaesthetics; therefore, differences in disease severity and previous treatment exposure may have influenced both response and clinical outcomes.

Substantial clinical and methodological heterogeneity was observed across studies. Ketamine dose, timing of initiation, infusion duration, concomitant therapies, seizure phenotype, and response definitions varied considerably. Outcomes ranged from partial reduction in seizure burden to complete or sustained electrographic cessation. This variability prevented meta-analysis, meaningful subgroup comparisons, and identification of an optimal dose, treatment duration, or therapeutic window.

Ketamine was rarely administered independently. Frequent coadministration of anaesthetic agents and additional antiseizure medications limited attribution of seizure resolution or haemodynamic changes specifically to ketamine. Baseline cardiovascular instability, vasopressor exposure, and modifications to concomitant therapy were also inconsistently reported.

Finally, several clinically relevant variables, including sex, aetiology, functional outcomes, and long-term neurological recovery, were incompletely or inconsistently described. Survival was assessed at different time points, further limiting comparisons. The restriction to English- and Spanish-language publications may also have introduced language bias, while reporting bias and certainty of evidence could not be formally assessed because of the small number of studies and heterogeneous outcomes.

## Conclusion

Ketamine-containing regimens were associated with clinical or electrographic improvement in a substantial proportion of patients with refractory or super-refractory status epilepticus, while immediate haemodynamic deterioration appeared uncommon in the reported studies. However, the available evidence remains limited by predominantly observational designs, substantial heterogeneity in dosing and timing, inconsistent response definitions, and frequent concomitant use of other antiseizure medications and intravenous anaesthetics. Consequently, the independent effect of ketamine on sustained seizure control, survival, and functional recovery cannot yet be established. Are needed clinical trial and prospective studies to define optimal timing, dosing, safety profile, and clinical effectiveness of ketamine in RSE and SRSE therapy.

## Data Availability

The original contributions presented in the study are included in the article/Supplementary Material, further inquiries can be directed to the corresponding author/s.
